# Process Evaluation of a Psychosocial Intervention Addressing Women in a Disadvantaged Setting

**DOI:** 10.5539/gjhs.v4n1p22

**Published:** 2012-01-01

**Authors:** Rima Nakkash, Loulou Kobeissi, Zeina Ghantous, Maya Abou Saad, Brigitte Khoury, Nasser Yassin

**Affiliations:** Center for Research on Population and Health Department of Health Promotion and Community Health Faculty of Health Sciences, American University of Beirut Beirut, Lebanon; UCLA-School of Public Health Community Health Sciences Department Center for Research on Population and Health Epidemiology and Population Health Department Faculty of Health Sciences- American University of Beirut, Beirut, Lebanon Tel: 1-248-787-3427 E-mail: loulou.kobeissi@gmail.com; Center for Research on Population and Health Epidemiology and Population Health Department Faculty of Health Sciences, American University of Beirut, Beirut, Lebanon; Faculty of Medicine, Department of Psychiatry American University of Beirut, Beirut, Lebanon; Epidemiology and Population Health Department, Faculty of Health Sciences, American University of Beirut, Beirut, Lebanon

**Keywords:** Process Evaluation, Psycho-social intervention, Disadvantaged Population, Hey el Selloum

## Abstract

**Objectives:**

This paper presents the process evaluation of a community-based randomized psycho-social trial aimed to enhance reproductive and mental health outcomes of disadvantaged women living in the southern suburb of Beirut-Lebanon. Process evaluation of public health interventions involves the monitoring and documentation of interventions’ implementation to allow for better understanding of planned outcomes and of intervention effectiveness.

**Methods:**

A community-based randomized trial was conducted. The intervention consisted of 12 sessions (of combined 30 minutes of relaxation exercises and 75 minutes of structured support groups) delivered twice per week over a period of six-weeks. A process evaluation was conducted during the implementation of the intervention. This process evaluation aimed to ensure that the intervention was delivered and implemented as planned, as well as to monitor women’s satisfaction and attendance. The main components of the process evaluation included: dose delivered, dose received, and reach. Closed ended questionnaires were administered before/after/during each intervention session. Data was entered and analyzed using SPSS. Analysis revolved around simple frequency distribution for categorical variables and means (SD) for continuous variables. Limited bivariate analysis (using CHI Square and Anova) was done.

**Results:**

Results indicated that the delivery, implementation, and reach of the intervention were favorable. Participation was acceptable and satisfaction rates were very high.

**Conclusion:**

These favorable findings pertaining to intervention satisfaction, reach and participation highlight a number of lessons for future intervention studies in the context of disadvantaged settings. They also support the importance of involving the local community members in intervention planning, implementation and evaluation early on. We believe that the community involvement in this trial directly and significantly contributed to the results of this process evaluation.

## 1. Introduction

Process evaluation of public health interventions involves the monitoring and documentation of interventions’ implementation to allow for better understanding of planned outcomes and intervention effectiveness. Possible challenges to intervention implementation could include low participation and attendance, as well as discrepancy in delivery of what was planned as an intervention (dose intended), and what was delivered or received by participants (actual dose received) (Johnson *et al.*, 2009). Process evaluation findings could provide confirmation that the program activities are associated with the outcomes observed (McDonald, 2009). The literature provides numerous frameworks for process evaluation, one of which: is Linnan and Steckler’s, 2002. This framework outlines the basic elements of a comprehensive process evaluation plan, which include: a measure of fidelity, dose delivered, dose received, reach, recruitment, and context. *Fidelity* is “the extent to which the intervention was delivered as planned” (Oude Hengel *et al.*, 2010, p.5). *Dose delivered* is how much of the intended intervention was delivered as planned. *Dose received* is satisfaction or the “extent to which participants actively engage with, interact with, are receptive to, and/or use materials or recommended resources” (Linnan & Steckler, 2002, p.12). *Reach* is defined as “the degree to which the intended audience participates in an intervention” (Linnan & Steckler, 2002, p.12). *Context* includes characteristics of the environment that may affect implementation development including the physical, social, and political environment (Linnan & Steckler, 2002 p. 12).

This paper will provide a review of the findings of the process evaluation of a community-based randomized psycho-social trial, aimed to enhance reproductive and mental health outcomes of disadvantaged women living in the southern suburb of Beirut-Lebanon. This process evaluation will report on dose delivered, dose received, and reach (the measured process evaluation components as agreed by the research team). Although, there is a multiplicity of public health programs conducted among women by various groups in Lebanon, to our knowledge, this is the first peer reviewed article on process evaluation of an intervention among disadvantaged women in a low income setting in Beirut, Lebanon. This paper will also provide a description of the intervention context, content, evaluation data collection tools, and finally report on process evaluation findings.

## 2. Methods

### 2.1 Description of the Context

The intervention setting was in Hay el Sellom (HES), in the southern suburbs of Beirut, the capital of Lebanon. HES is an informal settlement, stretched over an area of less than 1 Km^2^ and housing approximately 100,000 to 150,000 inhabitants. During the Lebanese Civil war period, people flocked into the area, which was originally a farming land and built concrete houses with no consideration to urban planning, building laws or basic infrastructure. Due to the prolongation of the Lebanese civil strife, the people forcedly stayed in this area, seeking security and employment. As a result of this unplanned movement, the sporadic and illegal construction resulted in insufficient basic physical infrastructure- thus creating a relatively crowded neighborhood. Despite the end of the civil war, the government still does not recognize the area in legal terms, and continues to pay little or no attention to providing it with public services (Kobeissi *et al.*, 2011; Makhoul, 2003).

### 2.2 Description of the intervention

The trial aimed to test the impact of a psychosocial intervention package (of combined 30 minutes of relaxation exercises and 75 minutes of structured support groups delivered twice per week over six weeks) on the reporting of medically unexplained vaginal discharge (MUVD)- and common mental disorders (known as CMDs: namely anxiety and/or depression). The package was conducted among 196 currently married women, 18-49 years of age with reported symptoms of MUVD and low to moderate levels of CMDs. Ninety nine women were randomized into the intervention group and 97 were randomized into the control group (Kobeissi *et al.*, 2011).

The components and the duration of this intervention package were guided by evidence-based literature supporting the positive impacts of structured social support on disease (Targ & Levine, 2002; Telch & Telch, 1986; Fung & Chien, 2002, Manandhar *et al.*, 2004; Bryce, Stanley & Graner, 1991; Panzarella *et al.*, 2006; Lumley *et al.*, 2006; Dennis, 2005; Constantino *et al.*, 2005; Scheidlinger & Kahn, 2005; Wiggins, 2004) and mental health outcomes (Magliano *et al.*, 2002; Araya *et al.*, 2003; Sumathipala *et al.*, 2000; Chen *et al.*, 2001; Ning *et al.*, 2001; Li, 2001; Krawczynski & Olszewski, 2000; Constantino *et al.*, 2005; Scheidlinger & Kahn, 2005; Wiggins, 2004). Also a review of the literature supports the positive impacts of progressive muscle relaxation and/or guided imagery on mental health and persistent unexplained physical symptoms (Kominars, 1997; Baird & Sands, 2004; Crook *et al.*, 1998; Baider *et al.*, 2001; Carlson & Bultz, 2004). The main aim behind this trial was to develop a simple low cost community-based intervention that has the potential to be later delivered, and easily sustained, at the level of the local community centres (if results deemed favourable).

### 2.3 The intervention Content

The intervention package consisted of 12 sessions implemented over a six week period using the facilities of the Lebanese Ministry of Social Affairs (MOSA) local center in HES). The 75 minutes semi-structured social support sessions (SSS) were run by masters’ level psychologists who moderated the sessions and were assisted by social workers as co-moderators. The SSS sessions were divided into directed and semi-structured social support discussion sessions, incorporating problem solving skills building training coupled with venting (Table 1). To ensure coherence among group members, the group was closed; meaning that once a group was formed its members remained in the same group throughout the intervention period. A manual was developed by a senior doctorate level clinical psychologist highlighting the content of each of the 12 sessions and was pilot-tested with women of similar characteristics. This manual was delivered to the moderators and co-moderators during training sessions, where it was modified and finalized accordingly with the team.

**Table 1:** Overview of intervention package

Social Support Component	General outline of Content
First two sessions	Introductions and agreement on group rules, ice breaking
Sessions 3-23	Welcoming and attendance taking, getting feedback on the previous session, deciding on the topic of the day, facilitating discussion, teaching problem solving skills, and wrap up of session’s conclusions
Session 24	Concluding with a debriefing of gained skills and group experiences. It was accompanied with a farewell ceremony.
**Relaxation exercise component**	
Progressive muscle relaxation (PMR)	The program focuses on 16 muscle groups which are relaxed in a specified order and lasts for not more than 5 to six minutes to avoid over cooling of the body. Since it is a progressive technique, the relaxation is achieved over a long period of time.
Guided Imagery (GI)	People are coached to create calming, peaceful images to induce relaxation. A pleasant imagery from the past is imagined whereby the details of this image is recalled and relaxation during this imagination process is experienced.
Stretching and Breathing	Promotes relaxation and reduces stress Breathing techniques usually accompany stretching exercises to achieve the desired effect.
Progressive Resistance training (PR)	Progressive resistance is a training that improves muscular fitness. PR is characterized by gradual increases in resistance (weight) over a certain period of time leading in muscular strength and or endurance exp. Weight training is the type of progressive resistance that is highly suggested for improving muscular fitness where using elastic bands can be a part of it.

The 30 minutes **relaxation exercise (RE)** sessions were run by physical trainers. The relaxation exercises revolved around the delivery of four main components: progressive resistance training, progressive muscle resistance, stretching and breathing, and guided imagery (Table 1). These components were introduced into the sessions gradually, starting at 15 minutes with easy complexity and reaching up to 30 minutes with a relatively harder complexity (by the fourth session). These exercises also included teaching women how to engage in visual guided imagery exercises on their own at home. The sessions were intentionally progressive in nature in order to properly build up resistance. Each new session of the RE package would start with a wrap up about the techniques conducted in the previous sessions, touch at what is being practiced at home and proceed by adding an additional new technique. A manual was also developed by a physical fitness specialist who supervised and trained the physical trainers during a two-day training workshop. The manual was provided for the physical trainers as a guide on each of the sessions. In addition, a brief pamphlet was given to women participants to describe the components of each session.

The implementation and evaluation team included: five clinical psychologists, five social workers, five physical trainers, five process evaluators recruited from the local women’s committee (LWC), a field assistant and two supervisors.

### 2.4 Data Collection Tools

Process evaluation analysis revolved around: Dose delivered, dose received and reach. Process evaluation data was collected from each session using developed data collection tools to capture the desired components (Table 2). These tools were mainly close-ended questionnaires delivered either before the start of the session to monitor attendance, during the session to assess dose received and reach and at the end of the session to assess satisfaction. All women in the sessions were administered the evaluation tools (questionnaires) by the members of the LWC and trainers who were trained on data collection. The LWC and the trainers were responsible for this assessment in the RE component of the intervention; while, the co-moderators were responsible for this assessment in the SSS. The co-moderator was assigned for this task in the SSS, in order to maintain the privacy and confidentiality of issues discussed within a group. Hence, no outside evaluator was assigned. These different data collection tools (questionnaires) were pretested during the pilot phase and adjustments were made, as suggested by the LWC and the trainers, accordingly. For example, in the RE component, when rating the extent to which activities were implemented as planned, the initial questionnaire had listed in detail every muscle that the exercise suggested to cover in each session. The LWC thought it was not feasible to evaluate to that level of detail. The evaluation questionnaire was adjusted accordingly and required only noting overall if suggested exercises were implemented or not. One member of the implementation team was assigned to follow-up on the process evaluation, to ensure all forms were filled in due time, and to collect completed forms at the end of each session.

**Table 2:** Process evaluation component and description of corresponding data collection tools

PE component	Form	Description
Reach	A-Attendance	Attendance was taken at the beginning of each session, noting late arrivals and women who left early, if any. Each woman was provided with an ID number to accompany her throughout the intervention period.
Dose received	B-Women Satisfaction	Following each session, the evaluators (the LWC in RE and the co-moderator in SSS) asked the women to indicate on a sheet on a scale of one to ten the extent of satisfaction with the session; with one being the *least* satisfied and ten the *most* satisfied.
	C-Participation	Following each RE session, based on their observation, the trainer and LWC noted the extent of participation and involvement of the women. Following each of the SSS sessions, the moderator filled in a similar evaluation.
Dose delivered	D-Intervention delivery	Following each session, for the relaxation exercise, the trainer and LWC and for the SSS, the moderator and co-moderator evaluated the extent to which the activities were implemented as planned in the manual.

This trial gained ethical approval by the institutional review board of the American University of Beirut. Two different consent forms were used in the intervention trial: recruitment/baseline assessments and post randomization/post assessments. The first consent form was used during the study recruitment phase to check whether or not the woman conforms with the study inclusion criteria, particularly with respect to the reporting of the reproductive health and common mental distress outcomes. This consent form was read in private to the women orally by an interviewer and in front of a witness prior to examination.

The second consent form was administered to women who were found eligible to take part in the trial. The purpose of this consent form is to make sure that the women understand the randomization process and the reasons behind their selection in the trial. It also made sure that the women agree to comply throughout the trial’s period, to being randomized into either the intervention or the control group, and do to having repeated post assessments, clinical exams and laboratory tests. The interviewer explained the consent form to the women privately, which marked the initiation of the intervention trial.

### 2.5 Analysis

The data was entered by trained personnel using the Statisitical Package for Social Sciences (SPSS) software. Data entry error checks were made by randomly selecting 10% of the questionnaires. An independent research assistant was responsible for undertaking the error checks. Analysis revolved around simple frequency distribution for categorical variables and means (Standard Deviation (SD)) for continuous variables. Limited bivariate analysis (using CHI Square test and Anova) was also done.

## 3. Results

With regards to ***dose received***, in the evaluation of the RE component, trainers and LWC reported 98% and 94% of the sessions as positive and active, respectively. In the SSS components, the co-moderators reported 88% of the sessions as positive and active. With regards to women’s participation rate in the RE component, the trainers and LWC reported 100% and 97% respectively. In the SSS component, the co-moderators reported 100% participation (Table 3).

**Table 3:** Dose received. Participation

	% women rating the session as positive and active		% of active participation	
Data collected by :				
***Relaxation exercise***	Trainer	LWC	Trainer	LWC
All Some	97.5 2.5	94.2 2.5	100	96.7
Data collected by :				
***SSS***	Co-moderator	Moderator	Co-Moderator	Moderator
All Some	88.3 11.7	*--*	100	*-*

* *n= 120 sessions*

**Figure 1:**
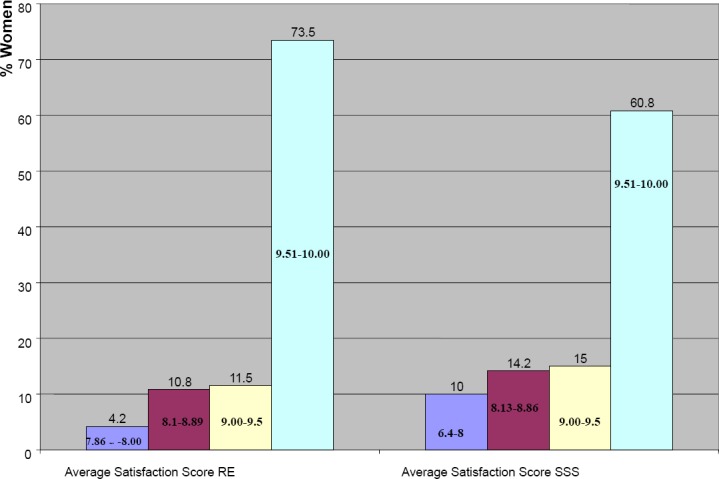
Satisfaction

For the Relaxation exercise sessions and the SSS sessions, 76% of women had had an over all satisfaction score of 10. (Figure 1)

With regards to ***dose delivered***, in the evaluation of the RE component, trainers reported 97% and LWC 99% of the sessions were completed as per the manual. In the SSS component the co-moderator rated 93% and moderator 99% sessions completed as per manual (Table 4).

**Table 4:** Dose delivered. Intervention delivery

% Session completed as per manual		
Data collected by		
***Relaxation exercise***	Trainer	LWC
Fully Partially	96.7 0.8	99.2 0.8
Data collected by		
***SSS***	Co-moderator	Moderator
Fully Partially	93.3 6.7	99.2 0.8

* *n= 120 sessions*

**Figure 2:**
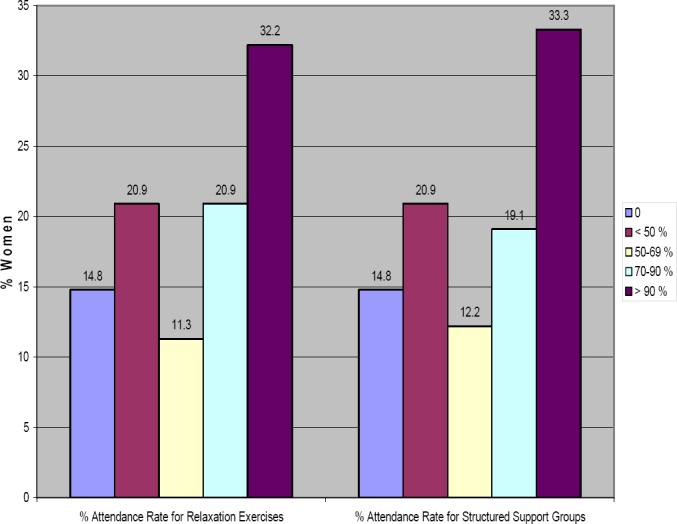
REACH: Attendance Rate (% Sessions Attended)

In terms of reach, in the RE component 32.2% of women attended 90% of the sessions, 20.9% of the women attended 70-90% of the sessions, 11.3% attended 50-69%, 20.9 % attended less than 50%, and 14.8% none of the sessions (Figure 2). A similar trend of attendance was observed in the SSS sessions. This is expected since the sessions were consecutive to one another, thus, if one woman attended one it was likely that she would also stay for the following one.

## 4. Discussion

The objective of this process evaluation was to ensure that the intervention was delivered and implemented as planned as well as to monitor women’s satisfaction and attendance. Overall, process evaluation indicated favorable results pertaining to the delivery and implementation of the intervention as intended and planned. Over 90% of the intervention dose was delivered as outlined in the manual. Sessions were rated as positive and active, and the women were evaluated to have actively participated in the delivered sessions. Thus, participation and satisfaction was very high. Similarly, satisfaction rates were high for each of the two components of the intervention package. Intervention reach, on the other hand, was acceptable. Up to 33% of the women actually attended all 12 sessions. This is contingent with results of studies on process evaluation reported elsewhere on other public health interventions (Anderson, 1985; Dynes *et al.*, 2011).

A review of the literature did not result in papers reporting on process evaluations of women of similar age group and intervention. The published literature reports on process evaluations of a variety of public health interventions and varies in the measures used for process evaluation. Anderson (1985), in an evaluation of a psychological intervention among 60 year old or older women (in an aim to decrease loneliness in its social and emotional dimensions), reported 28.6% of the women (total study sample was 64 women) attending all for intervention meetings. This article, however, does not include data on dose received or delivered. A process evaluation of a maternal child health programme that involved a home-based life saving skills program (in an attempt to reduce mortality rates among mothers and children) in Bangladesh reported that over 51% of pregnant women attended all meetings (total study sample was 4500 women) (Dynes *et al.*, 2011). The largest percent (85.5%) received 2 sessions, followed by around 70% who received three sessions (Dynes *et al.*, 2011).

With regards to dose delivered, a paper reporting on evaluating nurses’ implementation of an infant-feeding counseling protocol for HIV-infected mothers: The Ban Study in Lilongwe, Malawi, reported that all 6 nurses implemented the protocol at an acceptable level of 90% implementation adherence or above. This study reported only on implementation but did not include measures of satisfaction, participation, or reach (Ferguson *et al.*, 2009).

Our process evaluation findings reveal acceptable attendance rates (> 50% of the women attended 70% of the sessions or more). Reasons given for non-attendance by the women were mostly in line with what the literature cites. In terms of reasons, women mainly responded: sickness in the family death of family member, family commitment (relatives visiting for leisure/vacation), husbands’ refusal, work, the intervention locale was far from their houses, or refusal to participate because of the lack of financial benefits. The literature provides a review of challenges to compliance in interventions, which could include: lack of follow-up and contact with subjects, women’s employment (Yancey *et al.*, 2006), family obligations, lack of proper telephone for communication (Beckie *et al.*, 2009). This is consistent with the reasons we found out during intervention delivery due to lack of attendance.

However, despite these barriers, attendance was considered acceptable, particularly given the uniqueness of the trial’s location: a disadvantaged setting in the suburbs of the capital. We believe that the implemented community based-participatory approach played a significant role in actively mobilizing the different members of the community. This has strengthened peoples’ trust and rapport with the research team, and in turn rendered high commitment to attendance. Working from within was the guiding principle behind implementing this work. Employing a women’s committee, whose members were local women, through out the three years of the trial (and since the inception of the project proposal), also equally and significantly rendered the delivery of this intervention successful, and well received at the community level. Further to this, the timing of the delivery of the intervention took into account women’s time. The sessions were intentionally delivered during the mornings (between 8am- 12:00 pm) allowing time for the children to go to school as well as giving a leeway to go back home for house chores before the children came back from school. Child care at the site of the intervention was also provided in order to accommodate for women with new born babies as well as with little children. In order to further entice women, little incentives were also provided such as training suits (in order to make the less economically privileged women feel at ease about their attire) and incorporating music during the relaxation exercises, which was very well received by participants.

Similar adopted strategies to increase attendance are noted in the literature. For example, Sears *et al*. (2003) suggested the importance of providing transportation, childcare, flexible appointment times as a way to avoid attendance associated problems. Also, distance between home and intervention locations (Oleson *et al.*, 2008) as well as timings (Fredman et. al., 2009) were likely cited barriers to attendance.

A number of lessons for future intervention studies in disadvantaged settings could be learnt from our experience. Our findings support the importance of involving the local community in intervention planning and implementation early on- as we believe this was critical and significantly contributed to the high satisfaction rates as well as our acceptable attendance rate. Involvement of local women in process evaluation gave the study credibility and full trust by the participating women. It also allowed for better communication of objectives and plans. The proximity of the intervention locale to women’s homes also meant that they did not have to worry about transportation. The location was both convenient and familiar to participating women. Findings that showed high levels of participation and satisfaction provide a good evaluation of the trainers implementing the intervention. The process evaluation documented the appropriate delivery of the intervention in terms of dose received and dose reached. Findings will help interpret the outcome data.

## References

[ref1] Anderson L (1985). Intervention against loneliness in a group of elderly women: An impact evaluation. Social Science & Medicine.

[ref2] Araya R, Rojas G, Fritsch R (2003). Treating depression in primary care in low-income women in Santiago, Chile: A randomized controlled trial. Lancet.

[ref3] Baird C. L, Sands L (2004). A pilot study of the effectiveness of guided imagery with progressive muscle relaxation to reduce chronic pain and mobility difficulties of osteoarthritis. Pain Management Nursing.

[ref4] Baider L, Peretz T, Hadani P. E (2001). Psychological intervention in cancer patients: A randomized study. General Hospital Psychiatry.

[ref5] Beckie T. M, Mendonca M. A, Fletcher G. F (2009). Examining the Challenges of Recruiting Women into a Cardiac Rehabilitation Clinical Trial. Journal of Cardiopulmonary Rehabilitation & Prevention.

[ref6] Bryce R. L, Stanley F. J, Garner J. B (1991). Randomized controlled trial of antenatal social support to prevent preterm birth. British Journal of Obstetrics Gynaecology.

[ref7] Carlson L. E, Bultz B. D (2004). Efficacy and medical cost offset of psychosocial interventions in cancer care: Making the case for economic analyses. Psycho-oncology.

[ref8] Chen K. M, Synder M, Krichbaum K (2001). Tai chi and well-being of Taiwanese community-dwelling elders. Clinical Gerontologist.

[ref9] Constantino R, Kim Y, Crane P. A (2005). Effects of social support intervention on health outcomes in residents of a domestic violence shelter: A pilot study. Issues in Mental Health Nursing.

[ref10] Crook P, Rose M, Salmon P (1998). Adherence to group exercise: Physiotherapist-led experimental programmes. Physiotherapy.

[ref11] Dennis C. L (2005). Psychosocial and psychological interventions for prevention of postnatal depression: systematic review. Biomedical Journal.

[ref12] Dynes M, Rahman A, Beck D (2011). Home-based life saving skills in Matlab, Bangladesh: a process evaluation of a community-based maternal child health programme. Midwifery.

[ref13] Ferguson Y. O, Eng E, Bentley M (2009). Evaluating nurses’ implementation of an infant-feeding counseling protocol for HIV-infected mothers: The Ban Study in Lilongwe, Malawi. AIDS Education and Prevention.

[ref14] Fredman S. J, Baucom D. H, Gremore T. M (2009). Quantifying the recruitment challenges with couple-based interventions for cancer: applications to early-stage breast cancer. Psycho-Oncology.

[ref15] Fung W, Chien W (2002). The effectiveness of a mutual support group for family caregivers of a relative with dementia. Archives of Psychiatric Nursing.

[ref16] Johnson C. C, Myers L, Webber L. S (2009). A School-Based Environmental Intervention to Reduce Smoking among High School Students: The Acadiana Coalition of Teens against Tobacco (ACTT). International Journal of Environmental Research and Public Health.

[ref17] Kobeissi L, Nakkash R, Ghantous Z (2011). Evaluating a Community Based Participatory Approach to Research with Disadvantaged Women in the Southern Suburbs of Beirut. J Community Health.

[ref18] Krawczynski M, Olszewski H (2000). Psychological well-being associated with a physical activity programme for persons over 60 years old. Psychology of Sport & Exercise.

[ref19] Li F, Duncan T. E, Duncan S. C (2001). Enhancing the psychological well-being of elderly individuals through Tai Chi exercise: A latent growth curve analysis. Structural Equation Modelling: A Multidisciplinary Journal.

[ref20] Linnan L, Steckler A, Linnan L, Steckler A (2002). Process evaluation for public health interventions and research: an overview. Process Evaluation for Public Health Interventions and Research.

[ref21] Lumley J, Watson L, Small R (2006). PRISM (Program of Resources, Information and Support for Mothers): A community-randomised trial to reduce depression and improve women’s physical health six months after birth. Biomedical Journal of Public Health.

[ref22] Magliano L, Marasco C, Fiorillo A (2002). Working Group of the Italian National Study on Families of Persons with Schizophrenia: The impact of professional and social network support on the burden of families of patients with schizophrenia in Italy. Acta Physchiatrica Scandainvaica.

[ref23] Makhoul J (2003). Physical and social contexts of the three urban communities of Nabaa, the Bourj El Barajneh Palestinian camp and Haly Selloum. Centre for Reseach and Population Health. American University of Beirut (Unpublished Research Memo).

[ref24] Manandhar D. S, Osrin D, Shrestha B. P, Mesko, members of the MIRA Makwanpur trial team (2004). Effect of a participatory intervention with women’s groups on birth outcomes in Nepal: Cluster-randomised controlled trial. Lancet.

[ref25] McDonald S-K, Sykes G, Schneider B, Plank D. N (2009). Scale-Up as a Framework for Intervention, Program, and Policy Evaluation Research. Handbook of Education Policy Research.

[ref26] Oleson J. J, Breheny P. J, Pendergast J. F (2008). Impact of travel distance on WISEWOMAN Intervention attendance for a rural population. Preventive Medicine.

[ref27] Oude Hengel K. M, Joling C. I, Proper K. I (2010). A work site prevention program for construction workers: design of a randomized controlled trial. BioMed Central.

[ref28] Panzarella C, Alloy L. B, Whitehouse W. G (2006). Expanded hopelessness theory of depression: On the mechanisms by which social support protects against depression. Cognitive Therapy & Research.

[ref29] Scheidlinger S, Kahn G. B (2005). In the aftermath of September 11: group interventions with traumatized children revisited. International Journal of Group Psychotherapy.

[ref30] Sears S. R, Stanton A. L, Kwan L (2003). Recruitment and retention challenges in breast cancer survivorship research: results from a multisite, randomized intervention trial in women with early stage breast cancer. Cancer Epidemiology, Biomarkers & Prevention.

[ref31] Sumathipala A, Hewege S, Hanwella R (2000). Randomized controlled trial of cognitive behaviour therapy for repeated consultations for medically unexplained complaints: A feasibility study in Sri Lanka. Psychological Medicine.

[ref32] Targ E. F, Levine E. G (2002). The efficacy of a mind-body-spirit group for women with breast cancer: A randomized controlled trial. General Hospital Psychiatry.

[ref33] Telch C. F, Telch M. J (1986). Group coping skills instruction and supportive group therapy for cancer patients: a comparison of strategies. Journal of Consulting & Clinical Psychology.

[ref34] Wiggins M, Oakley A, Roberts I (2004). The social support and family health study: A randomised controlled trial and economic evaluation of two alternative forms of postnatal support for mothers living in disadvantaged inner-city areas. Health Technology Assessment.

[ref35] Yancey A. K, Ortega A. N, Kumanyika S. K (2006). Effective recruitment and retention of minority research participants. Annual Review of Public Health.

